# Two-Dimensional Covalent Organic Frameworks for Carbon Dioxide Capture through Channel-Wall Functionalization

**DOI:** 10.1002/anie.201411262

**Published:** 2015-01-22

**Authors:** Ning Huang, Xiong Chen, Rajamani Krishna, Donglin Jiang

**Affiliations:** Department of Materials Molecular Science, Institute for Molecular Science, National Institutes of Natural Sciences5-1 Higashiyama, Myodaiji, Okazaki 444-8787 (Japan); Van't Hoff Institute for Molecular Sciences, University of AmsterdamScience Park 904, 1098 XH Amsterdam (The Netherlands)

**Keywords:** carbon dioxide, covalent organic frameworks, flue gas separation, gas adsorption, synthesis

## Abstract

Ordered open channels found in two-dimensional covalent organic frameworks (2D COFs) could enable them to adsorb carbon dioxide. However, the frameworks’ dense layer architecture results in low porosity that has thus far restricted their potential for carbon dioxide adsorption. Here we report a strategy for converting a conventional 2D COF into an outstanding platform for carbon dioxide capture through channel-wall functionalization. The dense layer structure enables the dense integration of functional groups on the channel walls, creating a new version of COFs with high capacity, reusability, selectivity, and separation productivity for flue gas. These results suggest that channel-wall functional engineering could be a facile and powerful strategy to develop 2D COFs for high-performance gas storage and separation.

Covalent organic frameworks (COFs), a class of crystalline porous polymers that allow the atomically precise integration of building blocks into periodicities, have emerged as a new platform for designing advanced organic materials with periodic structures.^1^–^5^ Two-dimensional (2D) COFs have limited surface areas and small pore volumes as a result of their dense π-stacking layer structure, which greatly restricts their potential as a porous medium for the adsorption of gases such as carbon dioxide, methane, and hydrogen. With the exception of two examples utilizing azine 2b and boronate 3d linkages that interact with specific gas molecules to exhibit good capacity, the majority of 2D COFs have very low performance in gas adsorption. To improve this situation, we present a strategy that explores the channel walls for functional engineering and demonstrate its significance and effectiveness in the design of 2D COFs for high-performance gas adsorption and separation.

The advantage of the dense layer structure of 2D COFs is that this architecture enables the dense incorporation of functional groups onto the channel walls. This structural benefit compensates for the low porosity of 2D COFs. We observed that functional engineering of the channel walls converts a conventional 2D COF into an outstanding carbon-dioxide-capture material. We demonstrated this strategy by using a conventional imine-linked 2D COF (Figure 1 a,b, [HO]_100 %_-H_2_P-COF) as a scaffold with porphyrin at the vertices and phenol units on the pore walls; this 2D COF exhibits a low capacity for carbon dioxide adsorption. The phenol groups undergo a quantitative ring opening reaction with succinic anhydride that decorates the channel walls with open carboxylic acid groups (Figure 1 a,c, [HO_2_C]_100 %_-H_2_P-COF). The content of carboxylic acid units on the channel walls was tuned by adjusting the content of phenol groups through a three-component condensation system with a mixture of 2,5-dihydroxyterephthalaldehyde (DHTA) and 1,4-phthalaldehyde (PA) as the wall components (Figure 1 a, [HO]_*X* %_-H_2_P-COFs, *X*=[DHTA]/([DHTA]+[PA])). Various analytic methods revealed that the DHTA-to-PA molar ratios integrated into [HO]_*X* %_-H_2_P-COFs were identical to those employed for the reactions (see the Supporting Information, SI, Table S1, Figures S1 and S2). Using this method, we synthesized a series of [HO_2_C]_*X* %_-H_2_P-COFs with controlled carboxylic acid density that varied from 25 % to 50 %, 75 %, and 100 % (Figure 1 a). The [HO_2_C]_*X* %_-H_2_P-COFs were characterized by using infrared spectroscopy (Figure S1), elemental and thermogravimetric analysis (Table S1, Figure S3), energy dispersive X-ray spectroscopy (Table S2, Figure S4), solution-state ^1^H NMR spectroscopy of hydrolyzed samples (Table S3, Figure S5), and X-ray diffraction (XRD) measurements. These methods show that the content of carboxylic acid units integrated into the channel walls is close to the *X* % value of [HO_2_C]_*X* %_-H_2_P-COFs (Table S3). Notably, compared to metal-catalyzed azide–ethynyl 2c,d or other click reactions, 4c this ring opening reaction is free of metal catalysts, proceeds smoothly and cleanly, and excludes the formation of metal nanoparticles that would contaminate the channels.

**Figure 1:**
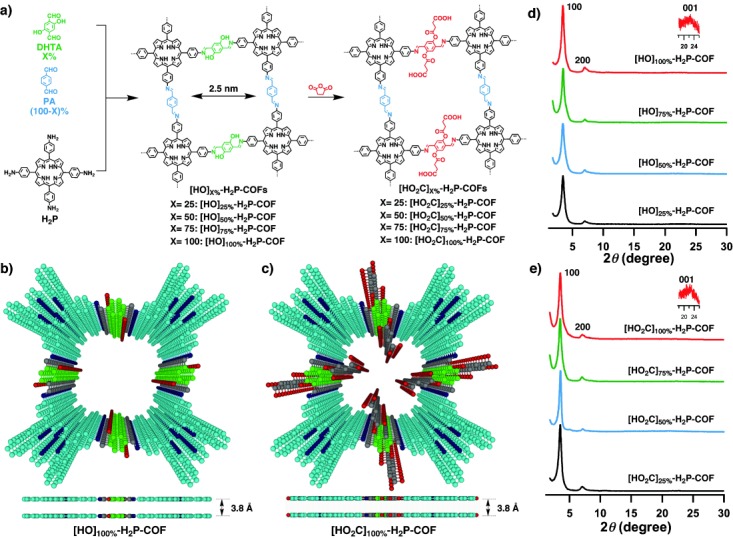
a) Synthesis of [HO_2_C]_*X* %_-H_2_P-COFs with channel walls functionalized with carboxylic acid groups through the ring opening reaction of [OH]_*X* %_-H_2_P-COFs with succinic anhydride. Top views of b) [HO]_100 %_-H_2_P-COF and c) [HO_2_C]_100 %_-H_2_P-COF. XRD patterns of d) [OH]_*X* %_-H_2_P-COFs and e) [HO_2_C]_*X* %_-H_2_P-COFs.

The [HO]_100 %_-H_2_P-COF samples exhibited strong XRD peaks at 3.49°, 7.21°, and 23.1°, which are assignable to 100, 200, and 001 facets, respectively (Figure 1 d, red curve). 2d Other [HO]_*X* %_-H_2_P-COFs exhibited identical diffraction patterns (Figure 1 d). The [HO_2_C]_*X* %_-H_2_P-COFs exhibit XRD patterns (Figure 1 e) similar to those of [HO]_*X* %_-H_2_P-COFs, which indicates that the crystal structure of [HO_2_C]_*X* %_-H_2_P-COFs is similar to that of [HO]_100 %_-H_2_P-COF. 2d The presence of (001) facet at 23.1°, which corresponds to an interlayer interval of 3.8 Å, indicates that [HO]_*X* %_-H_2_P-COFs and [HO_2_C]_*X* %_-H_2_P-COFs have dense π-stacking layer structures (Figure 1 b,c).

The crystal structures of [HO]_*X* %_-H_2_P-COFs and [HO_2_C]_*X* %_-H_2_P-COFs suggest the presence of open nanochannels (Figure 1 b,c). The [HO]_*X* %_-H_2_P-COFs exhibit typical type IV nitrogen sorption isotherm profiles indicative of mesoporous character (Figure S6a). Notably, the Brunauer–Emmett–Teller (BET) surface area (1054–1284 m^2^ g^−1^), pore volume (0.89–1.02 cm^3^ g^−1^), and pore size (2.5 nm) remained nearly unchanged (Table 1). Pore size distribution profiles revealed that these COFs possess a single type of mesopores (Figure S6b–e). Therefore, the three-component condensation reaction is reasonably concluded to allow for the integration of phenol groups in a controlled manner while retaining crystallinity and porosity.

**Table 1:** Porosity, CO_2_ uptake, and *Q*_st_ value of [HO]_*X* %_-H_2_P-COFs and [HO_2_C]_*X* %_-H_2_P-COFs.

H_2_P-COFs	S_BET_ [m^2^ g^−1^]	Pore size [nm]	Pore volume [cm^3^ g^−1^]	CO_2_ uptake [mg g^−1^] at 1 bar		*Q*_st_ [kJ mol^−1^]
				273 K	298 K	
[HO]_25 %_	1054	2.5	0.89	54	31	32.2
[HO]_50 %_	1089	2.5	0.91	46	34	29.4
[HO]_75 %_	1153	2.5	0.96	52	32	31.5
[HO]_100 %_	1284	2.5	1.02	63	35	36.4
[HO_2_C]_25 %_	786	2.2	0.78	96	58	38.2
[HO_2_C]_50 %_	673	1.9	0.66	134	67	39.6
[HO_2_C]_75 %_	482	1.7	0.54	157	72	41.2
[HO_2_C]_100 %_	364	1.4	0.43	174	76	43.5

Functionalization of the channel walls with carboxylic acid groups triggers microporosity in [HO_2_C]_*X* %_-H_2_P-COFs, as evidenced by their typical type I sorption curves (Figure S6f). The BET surface area (*S*_BET_) decreased from 786 to 673, 482, and 364 m^2^ g^−1^, whereas the pore size decreased from 2.2 to 1.9, 1.7, and 1.4 nm (Table 1), as the content of carboxylic groups was increased from 25 % to 50 %, 75 %, and 100 %, respectively. The pore volume also decreased from 0.78 to 0.66, 0.54, and 0.43 cm^3^ g^−1^, as the content of carboxylic groups was increased. This reduction in porosity is indicative of space filling by the functional units appended to the channel walls. Notably, the pore size distribution profiles revealed that porosity was solely derived from the micropores (Figure S6g–j). This observation also suggests that the channel walls were randomly functionalized with carboxylic acid groups in the case of [HO_2_C]_*X* %_-H_2_P-COFs (*X*=25, 50, and 75). Stability tests showed that [HO_2_C]_*X* %_-H_2_P-COF was stable upon immersion in THF, water, and aqueous HCl (1 m), NaHCO_3_ (1 m), and KOH (1 m) solutions for 24 h (Figure S7).

Carboxylic acid groups have been reported to trigger a dipolar interaction with carbon dioxide. 6a–f In [HO_2_C]_*X* %_-H_2_P-COFs, the carboxylic acid units are located at the termini and exhibit an acidity similar to that of the free carboxylic acid, as evidenced by their p*K*_a_ value of 5.86. We first investigated the CO_2_ adsorption by [HO]_*X* %_-H_2_P-COFs at pressures up to 1 bar and at temperatures of 273 K (Figure S8a) and 298 K (Figure S8b). The [HO]_*X* %_-H_2_P-COFs exhibited low capacities between 46 and 63 mg g^−1^ at 273 K and between 31 and 35 mg g^−1^ at 298 K (Table 1). By contrast, the [HO_2_C]_*X* %_-H_2_P-COFs exhibited dramatically increased CO_2_ adsorption capacities. For example, [HO_2_C]_100 %_-H_2_P-COF exhibited a capacity of 180 and 76 mg g^−1^ at 273 K (Figure S8c) and 298 K (Figure S8d), respectively. These capacities are 2.8- and 2.2-fold greater than those of [HO]_100 %_-H_2_P-COF (Table 1). Interestingly, the adsorption capacity of [HO_2_C]_*X* %_-H_2_P-COFs increased in proportion to their carboxylic acid content (Figure S8c,d; Table 1). These positive effects clearly confirmed the effectiveness of channel-wall functionalization in enhancing CO_2_ adsorption.

Various 2D and 3D COFs with different structures have been previously synthesized and investigated in attempts to develop a practical scaffold for carbon dioxide adsorption. Typical examples include boronate-linked 2D COF-5 (5.9 wt %, *S*_BET_=1670 m^2^ g^−1^), 3c TDCOF-5 (9.2 wt %, *S*_BET_=2497 m^2^ g^−1^), 5a and 3D COF-103 (7.6 wt %, *S*_BET_=3530 m^2^ g^−1^), 3c imine-linked ILCOF-1 (6.0 wt %, *S*_BET_=2723 m^2^ g^−1^) 5b and TpPa-1 (15.6 wt %, *S*_BET_=535 m^2^ g^−1^), 3d and azine-linked ACOF-1 (17.7 wt %, *S*_BET_=1176 m^2^ g^−1^). 2b The capacity of [HO]_100 %_-H_2_P-COF (6.5 wt %) is close to those of conventional and nonfunctionalized COF-5, IL-COF-1, and COF-103. By contrast, the wall-channel functionalized [HO_2_C]_100 %_-H_2_P-COF takes up 4.1 mmol g^−1^ of CO_2_ (18.0 wt %, 180 mg g^−1^, *S*_BET_=364 m^2^ g^−1^), which is the highest performance among 2D and 3D COFs reported thus far. To the best of our knowledge, the capacity observed for [HO_2_C]_100 %_-H_2_P-COF is also comparable to those of other top-class members (Table S1), including PPN-6-SO_3_Li (187 mg g^−1^), 6g Amine-PCN-58 (128 mg g^−1^), 6p UCBZ-1 (99 mg g^−1^), 6q N-TC-EMC (176 mg g^−1^),^[6^ ^h]^ and PPN-6-CH_2_DETA (190 mg g^−1^). 6i

Upon functionalization with carboxylic acid groups that have affinity for carbon dioxide, [HO_2_C]_100 %_-H_2_P-COF may exhibit enhanced adsorption selectivity. Based on the CO_2_ and N_2_ sorption isotherm curves measured at 298 K (Figures S8 and S9), we investigated the selective adsorption of CO_2_ over N_2_, which is critical for carbon capture from air or flue gas streams. The ideal absorbed solution theory (IAST) of Myers and Prausnitz 7a is a well-established model for describing the adsorption of gas mixtures in porous materials. Using pure-component isotherm fits, we determined the adsorption selectivity defined by *S*_ads_=(*q*_1_/*q*_2_)/(*p*_1_/*p*_2_) using the IAST method (SI). The accuracy of the IAST calculations for estimating the component loadings for several binary mixtures in a wide variety of porous materials has been established by comparison with configurational-bias Monte Carlo (CBMC) simulations of mixture adsorption. We utilized [HO]_100 %_-H_2_P-COF and [HO_2_C]_100 %_-H_2_P-COF for the separation of a CO_2_/N_2_ mixture that is relevant for CO_2_ capture from flue gases and for our evaluation we assumed the CO_2_/N_2_ mixtures contained 15 % CO_2_ and 85 % N_2_, following the earlier work of Mason et al. 6l Figure 2 a and b show the IAST calculation of CO_2_ and N_2_ uptake capacities for the 15/85 CO_2_/N_2_ mixture at 298 K. Notably, [HO_2_C]_100 %_-H_2_P-COF exhibited a CO_2_ uptake capacity of 0.51 mol kg^−1^ at 100 kPa (=1 bar), whereas [HO]_100 %_-H_2_P-COF displayed an uptake of only 0.16 mol kg^−1^ (Figure 2 a). By contrast, [HO]_100 %_-H_2_P-COF exhibited an N_2_ uptake of 0.118 mol kg^−1^, which is substantially greater than that of [HO_2_C]_100 %_-H_2_P-COF (0.038 mol kg^−1^, Figure 2 b). These results clearly suggest that the functionalization of channel walls with carboxylic acid groups significantly enhances the CO_2_ adsorption capacity of the flue mixture gas. Figure 3 c presents the adsorption selectivity for the 15/85 CO_2_/N_2_ flue gas mixture, in comparison to those of CuBTC (a MOF), 6j MgMOF-74, 6k–n and NaX zeolite. 6n,o At low pressures, such as 0.1 kPa, the adsorption selectivity *S*_ads_ was 323, which is greater than that of both CuBTC (broken black curve) and NaX zeolite (broken blue curve) and is close to that of MgMOF-74 (broken green curve). By contrast, [HO]_100 %_-H_2_P-COF exhibited a selectivity of only 18 at 0.1 kPa. At 100 kPa, [HO_2_C]_100 %_-H_2_P-COF exhibited a selectivity of 77, whereas [HO]_100 %_-H_2_P-COF exhibited a selectivity of only 8. Notably, the selectivity of [HO_2_C]_100 %_-H_2_P-COF is sufficiently high for potential practical use.

**Figure 2:**
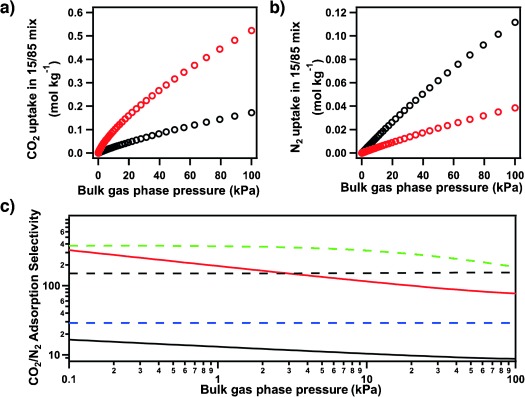
a) CO_2_ and b) N_2_ uptake by [HO]_100 %_-H_2_P-COF (black circles) and [HO_2_C]_100 %_-H_2_P-COF (red circles) of a 15/85 CO_2_/N_2_ flue gas mixture at 298 K. c) CO_2_/N_2_ absorption selectivity of [HO]_100 %_-H_2_P-COF (black curve) and [HO_2_C]_100 %_-H_2_P-COF (red curve) for the 15/85 CO_2_/N_2_ flue gas mixture at 298 K. The selectivities of NaX zeolite (broken blue curve), CuBTC (broken black curve), and MgMOF-74 (broken green curve) are shown for comparison.

**Figure 3:**
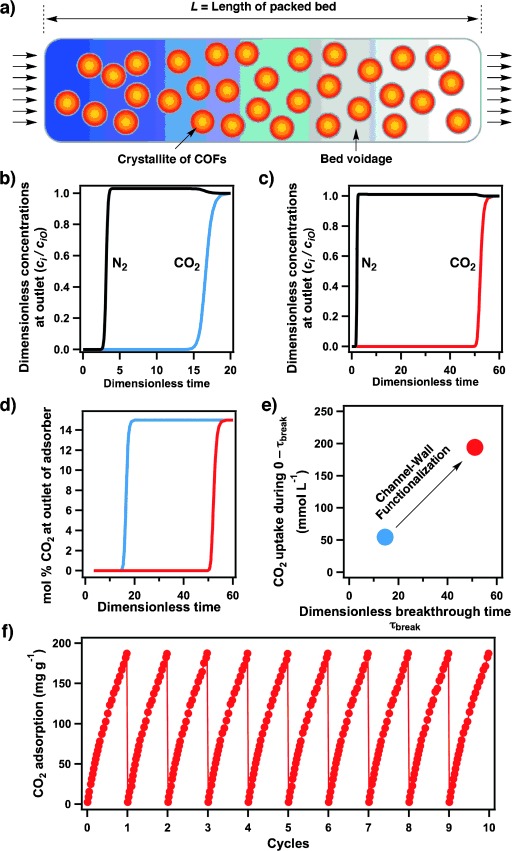
a) Fixed-bed adsorber for COFs. Flue gas breakthrough profiles of b) [HO]_100 %_-H_2_P-COF and c) [HO_2_C]_100 %_-H_2_P-COF at 298 K. d) Comparison of %CO_2_ at the adsorber outlet at 298 K (blue curve: [HO]_100 %_-H_2_P-COF, red curve: [HO_2_C]_100 %_-H_2_P-COF). e) Comparison of CO_2_ capture productivity at 298 K (blue spot: [HO]_100 %_-H_2_P-COF, red spot: [HO_2_C]_100 %_-H_2_P-COF). f) Cycle test of [HO_2_C]_100 %_-H_2_P-COF at 273 K.

To clarify the nature of CO_2_ adsorption, the isosteric heat of adsorption (*Q*_st_) was calculated from the CO_2_ adsorption isotherms measured at pressures up to 1 bar and at temperatures at 273 and 298 K. Interestingly, the *Q*_st_ value increased proportionally with the carboxylic acid content (Table 1). Therefore, the functionalized walls facilitate interactions with CO_2_ and contribute to the enhanced performance. The *Q*_st_ value was 43.5 kJ mol^−1^ for [HO_2_C]_100 %_-H_2_P-COF, which is compatible to that for MgMOF-74 and much higher than those for [HO]_100 %_-H_2_P-COF (34.5 kJ mol^−1^), CuBTC, and NaX zeolite.

To evaluate the gas separation ability of adsorbents under kinetic flowing gas conditions, breakthrough simulations were performed using a precise methodology established by Krishna and Long (SI). 7b These simulations properly reflect the separation capability of a pressure-swing adsorption (PAS) process, which is an energetically efficient method for industrial-scale capture. The performance of a COF in a PSA unit is governed by both selectivity and capacity factors. Figure 3 a presents a schematic of a packed-bed absorber. Figure 3 b and c show typical breakthrough curves for [HO]_100 %_-H_2_P-COF and [HO_2_C]_*X* %_-H_2_P-COF, respectively. The *x*-axis is dimensionless time, *τ*, defined as dividing the actual time, *t*, by the characteristic time, *Lε*/*μ* (SI). Clearly, [HO_2_C]_100 %_-H_2_P-COF exhibited a breakthrough time of 50, which is much longer than that of [HO]_100 %_-H_2_P-COF (15). Figure 3 d compares the breakthrough characteristics of COFs in terms of mol % CO_2_ at the outlet as a function of dimensionless time for operation at a total pressure of 100 kPa. [HO]_100 %_-H_2_P-COF (red curve) has a breakthrough time much longer than that of [HO]_100 %_-H_2_P-COF (blue curve). Longer breakthrough times are desirable for greater CO_2_ capture. For a quantitative evaluation of the COFs, we arbitrarily chose the required outlet gas purity to be <0.05 mol % CO_2_. Using this purity specification, we determined the breakthrough times, *τ*_break_, for each COF. On the basis of the material balance on the absorber, we determined the amount of CO_2_ captured during the time interval 0–*τ*_break_. Figure 3 e presents a plot of the number of mmol of CO_2_ captured per *L* of adsorbent during the time interval 0–*τ*_break_ against the breakthrough time *τ*_break_. Notably, [HO_2_C]_100 %_-H_2_P-COF (red circle) exhibited superior CO_2_ productivity compared with [HO]_100 %_-H_2_P-COF (black circle).

The aforementioned results indicate that channel-wall functionalization is efficient to convert a conventional COF into outstanding CO_2_ adsorption materials; the effects of functional groups on carbon dioxide capture are positive and profound ranging from capacity to selectivity and productivity. To examine the cycle performance of [HO_2_C]_100 %_-H_2_P-COF in terms of CO_2_ uptake, we conducted temperature and vacuum swings with a Belsorp mini II analyzer by saturating the samples with CO_2_ up to 1.0 bar at 273 K followed by placing the samples under high vacuum for 60 min at 353 K. Remarkably, after ten cycles, no significant decline in uptake capacity was observed (Figure 3 f), indicating complete desorption in each regeneration cycle and excellent cycling performance. These features assure a green process in regenerating the adsorbers.

In summary, we developed a strategy for converting a conventional 2D COF into an outstanding CO_2_ capture scaffold through channel-wall functionalization. The high-throughput ring opening reaction is useful for creating carboxylic-acid-functionalized channel walls while retaining the layered and open porous structure. Given the rather limited room for increasing the porosity of 2D COFs, together with the availability of a broad diversity of different functional groups, we anticipate that the present channel-wall engineering strategy will be critical to exploring 2D COFs for high-performance gas storage and separation.
