# Supplementary material for: Two-Dimensional Covalent Organic Frameworks for Carbon Dioxide Capture through Channel-Wall Functionalization
Source: Angew Chem Int Ed Engl. 2015 Jan 22;54(10):2986–90. doi: 10.1002/anie.201411262 (PMC4471552; doi:10.1002/anie.201411262)
Supplement: Supplementary file 1 [file anie0054-2986-sd1.pdf]

Angewandte Chemie
Eine Zeitschrift der Gesellschaft Deutscher Chemiker

Supporting Information

© Wiley-VCH 2015

69451 Weinheim, Germany

Two-Dimensional Covalent Organic Frameworks for Carbon Dioxide Capture through Channel-Wall Functionalization

*Ning Huang, Xiong Chen, Rajamani Krishna, and Donglin Jiang**

anie_201411262_sm_miscellaneous_information.pdf

Contents

Section A. Methods

Section B. Materials and synthetic procedures

Section C. Fitting of pure component isotherms

Section D. Isosteric heat of adsorption

Section E. IAST calculations

Section F. Simulation methodology for breakthrough in fixed bed absorbers

Section G. Notation

Section H. Supporting tables

Section I. Supporting figures

Section J. Supporting references

Section A. Methods

Fourier transform Infrared (FT IR) spectra were recorded on a JASCO model FT IR-6100 infrared spectrometer. UV-Vis-IR diffuse reflectance spectrum (Kubelka-Munk spectrum) was recorded on a JASCO model V-670 spectrometer equipped with integration sphere model IJN-727. Powder X-ray diffraction (PXRD) data were recorded on a Rigaku model RINT Ultima III diffractometer by depositing powder on glass substrate, from $2\theta = 1.5°$ up to 60° with 0.02° increment. Elemental analysis was performed on a Yanako CHN CORDER MT-6 elemental analyzer. TGA measurements were performed on a Mettler-Toledo model TGA/SDTA851^e under N_2, by heating to 800 °C at a rate of 10 °C min^{-1}. Nitrogen sorption isotherms were measured at 77 K with a Bel Japan Inc. model BELSORP-mini II analyzer. Before measurement, the samples were degassed in vacuum at 120 °C for more than 10 h. By using the non-local density functional theory (NLDFT) model, the pore volume was derived from the sorption curve. ^1H NMR spectra were recorded on a JEOL model JNM-LA400 NMR spectrometer, where the chemical shifts (δ in ppm) were determined with a residual proton of the solvent as standard. Scanning electron microscopy (SEM) was carried out using solid samples on Hitachi Hitechnology C C model SU6600 and energy dispersive X-ray spectrometry (EDX) was recorded on Bruker AXS model using Quantax system with XFlash 6|10 detector.

Section B. Materials and synthetic procedures

1-Butanol, *o*-dichlorobenzene (*o*-DCB), anhydrous acetone (99.5%), tetrahydrofuran, and acetic acid were purchased from Wako Chemicals. succinic anhydride and 1,4-phthalaldehyde (PA) was purchased from TCI. Free-base 5,10,15,20-tetrakis(p-tetraphenylamino) porphyrin (H_2P) was prepared from *p*-nitrobenzaldehyde using a literature procedure.[S1] 2,5-Dihydroxyterephthalaldehyde (DHTA) was synthesized according to a reported method.[S2]

$[HO]_{X\%}$-H_2P-COFs. An *o*-DCB/BuOH/6 M AcOH mixture (5/5/1 by vol.; 1.1 mL) of H_2P (0.02 mmol, 13.48 mg) and DHTA/PA (total 0.04 mmol) at different molar ratios of 25/75, 50/50, 75/25, and 100/0 was degassed in a Pyrex tube (10 mL) by three freeze-pump-thaw cycles. The tube was sealed off and heated at 120 °C for three days. The precipitate was collected by centrifugation, washed with anhydrous THF for five times, and washed with acetone twice. The powder was dried at 120 °C under vacuum overnight to give the corresponding product in isolated yields of 78%, 75%, 84%, and 82% for $[HO]_{25\%}$-H_2P-COF, $[HO]_{50\%}$-H_2P-COF, $[HO]_{75\%}$-H_2P-COF, and $[HO]_{100\%}$-H_2P-COF, respectively.

$[HO_2C]_{X\%}$-H_2P-COFs. $[HO]_{X\%}$-H_2P-COFs (30 mg) was weighed into a 10-mL glass vial, to which succinic anhydride (6 mL, 1.0 M solution in anhydrous acetone) was added. The reaction mixture was heated at 60 °C for two days. The precipitate was collected by centrifugation, washed with anhydrous THF for five times. The crude product was rinsed with THF for 48 h using a Soxhlet extractor. The powder was dried at 100 °C under vacuum overnight to give the corresponding products of $[HO_2C]_{25\%}$-H_2P-COF, $[HO_2C]_{50\%}$-H_2P-COF, $[HO_2C]_{75\%}$-H_2P-COF, and $[HO_2C]_{100\%}$-H_2P-COF, quantitatively.

Hydrolysis of $[HO_2C]_{X\%}$-H_2P-COFs. The $[HO_2C]_{X\%}$-H_2P-COFs (100 mg) sample were hydrolyzed by refluxing in a THF/H_2O (2/1 by vol., 24 mL) solution of KOH (3 M) for 5 days. After filtration, 5 mL of aqueous HCl solution (6 M) was added slowly to the filtrate and the mixture was stirred for 1 h. The greenish porphyrin precipitate was removed by filtration. The filtrate was evaporated under vacuum and submitted to ^{1}H NMR spectroscopy in d_6-DMSO. The content of carboxylic acid was calculated by using the proton integrates (Table S3, Figure S5).

Section C. Fitting of pure component isotherms

The salient properites of two different COFs ($[HO]_{100\%}$-H_2P-COF and $[HO_2C]_{100\%}$-H_2P-COF) are specified in Table 1. The potential of these COFs are evaluated for the separation of CO_2/N_2 mixtures that is relevant for CO_2 capture from flue gases. For our evaluations, we assume the CO_2/N_2 mixtures to contain 15% CO_2, and 85% N_2, following the earlier work of Mason et al.[S1]

The experimentally measured excess loadings of CO_2, and N_2, obtained at different temperatures, were first converted to absolute loadings before data fitting. The procedure for converting to absolute loadings is the same as described in the Supporting Information accompanying the paper of Wu et al.[S2] For the purpose of converting to absolute loadings, the pore volumes used are specified in Table S4. The isotherm data for CO_2 were fitted with the Langmuir-Freundlich model:

$$q = q_{sat} \frac{bp^{v}}{1 + bp^{v}} \qquad (1)$$

with T-dependent parameter b

$$b_A = b_0 \exp\left(\frac{E}{RT}\right) \qquad (2)$$

The Langmuir-Freundlich parameters for adsorption of CO_2 are provided in Table S5. The simpler Langmuir model was adequate for fitting the isotherm data for N_2; Table S6 provides the T-dependent Langmuir parameters for N_2 in different materials.

Section D. Isosteric heat of adsorption

The isosteric heat of adsorption, Q_{st}, defined as

$$Q_{st} = RT^2 \left(\frac{\partial \ln p}{\partial T} \right)_q \qquad (3)$$

was determined using the pure component isotherm fits using the Clausius-Clapeyron equation.

Section E. IAST calculations

The selectivity of preferential adsorption of component 1 over component 2 in a mixture containing 1 and 2, perhaps in the presence of other components too, can be formally defined as

$$S_{ads} = \frac{q_1/q_2}{p_1/p_2} \quad (4)$$

In equation (4), q_1 and q_2 are the *absolute* component loadings of the adsorbed phase in the mixture. These component loadings are also termed the uptake capacities. In all the calculations to be presented below, the calculations of q_1 and q_2 are based on the use of the Ideal Adsorbed Solution Theory (IAST) of Myers and Prausnitz.[S3] The accuracy of the IAST calculations for estimation of the component loadings for several binary mixtures in a wide variety of zeolites, and MOFs has been established by comparison with Configurational-Bias Monte Carlo (CBMC) simulations of mixture adsorption.[S4-S9]

Section F. Simulation methodology for transient breakthrough in fixed bed absorbers

The separation of CO_2/N_2 mixtures is commonly carried out in fixed bed absorbers in which the separation performance is dictated by a combination of three separate factors: (a) adsorption selectivity, (b) uptake capacity, and (c) intra-crystalline diffusivities of guest molecules within the pores. Transient breakthrough simulations are required for a proper evaluation of MOFs; the simulation methodology used in our work is described in earlier publications.[S10,S11] A brief summary of the simulation methodology is presented below.

Assuming plug flow of an *n*-component gas mixture through a fixed bed maintained under isothermal conditions (see schematic in Figure 4a), the partial pressures in the gas phase at any position and instant of time are obtained by solving the following set of partial differential equations for each of the species *i* in the gas mixture.[S12]

$$\frac{1}{RT}\frac{\partial p_i(t,z)}{\partial t} = -\frac{1}{RT}\frac{\partial \left(v(t,z)p_i(t,z)\right)}{\partial z} - \frac{\left(1-\varepsilon\right)}{\varepsilon}\rho\frac{\partial \overline{q}_i(t,z)}{\partial t}; \quad i = 1,2,\ldots n \qquad (5)$$

In equation (5), *t* is the time, *z* is the distance along the adsorber, *r* is the framework density, *e* is the bed voidage, *v* is the interstitial gas velocity, and $\overline{q}_i(t,z)$ is the *spatially averaged* molar loading within the crystallites of radius r_c, monitored at position *z*, and at time *t*.

At any time *t*, during the transient approach to thermodynamic equilibrium, the spatially averaged molar loading within the crystallite r_c is obtained by integration of the radial loading profile

$$\overline{q}_i(t) = \frac{3}{r_c^3}\int_0^{r_c} q_i(r,t)r^2\,dr \qquad (6)$$

For transient unary uptake within a crystal at any position and time with the fixed bed, the radial distribution of molar loadings, q_i, within a spherical crystallite, of radius r_c, is obtained from a solution of a set of differential equations describing the uptake

$$\frac{\partial q_i(r,t)}{\partial t} = -\frac{1}{\rho}\frac{1}{r^2}\frac{\partial}{\partial r}\left(r^2 N_i\right) \qquad (7)$$

The molar flux N_i of component *i* is described by the simplified version of the Maxwell-Stefan equations in which both correlation effects and thermodynamic coupling effects are considered to be of negligible importance[12]

$$N_i = -\rho D_i \frac{\partial q_i}{\partial r} \qquad (8)$$

Summing equation (**6**) over all n species in the mixture allows calculation of the *total average* molar loading of the mixture within the crystallite

$$\overline{q}_t(t,z) = \sum_{i=1}^{n} \overline{q}_i(t,z) \qquad (9)$$

The *interstitial* gas velocity is related to the *superficial* gas velocity by

$$v = \frac{u}{\varepsilon} \qquad (10)$$

In industrial practice, the most common operation uses a step-wise input of mixtures to be separated into an absorber bed that is initially free of adsorbents, i.e. we have the initial condition

$$t = 0; \quad q_i(0,z) = 0 \qquad (11)$$

At time $t = 0$, the inlet to the absorber, $z = 0$, is subjected to a step input of the n-component gas mixture and this step input is maintained till the end of the adsorption cycle when steady-state conditions are reached.

$$t \geq 0; \quad p_i(0,t) = p_{i0}; \quad u(0,t) = u_0 \qquad (12)$$

where u_0 is the superficial gas velocity at the inlet to the absorber.

The breakthrough characteristics for any component is essentially dictated by two sets of parameters: (a) The characteristic contact time $\frac{L}{v} = \frac{L\varepsilon}{u}$ between the crystallites and the surrounding fluid phase, and (b) $\frac{D_i}{r_c^2}$, that reflect the importance of intra-crystalline diffusion limitations. It is common to use the dimensionless time, $\tau = \frac{tu}{L\varepsilon}$, obtained by dividing the actual time t, by the characteristic time, $\frac{L\varepsilon}{u}$ when plotting simulated breakthrough curves.[11]

If the value of $\frac{D_i}{r_c^2}$ is large enough to ensure that intra-crystalline gradients are absent and the entire crystallite particle can be considered to be in thermodynamic equilibrium with the surrounding bulk gas phase at that time t, and position z of the adsorber

$$\overline{q}_i(t,z) = q_i(t,z) \qquad (13)$$

The molar loadings at the *outer surface* of the crystallites, i.e. at $r = r_c$, are calculated on the basis of adsorption equilibrium with the bulk gas phase partial pressures p_i at that position z and time t. The adsorption equilibrium can be calculated on the basis of the IAST. The assumption of

thermodynamic equilibrium at every position z, and any time t, i.e. invoking Equation (13), generally results in sharp breakthroughs for each component. Sharp breakthroughs are desirable in practice because this would result in high productivity of pure products. Essentially, the influence of intra-crystalline diffusion is to reduce the productivity of pure gases. For all the breakthrough calculations reported in this work, we assume negligible diffusion resistances for all materials and we invoke the simplified Equation (13).

Section G. Notation

b_A dual-Langmuir-Freundlich constant for species *i* at adsorption site A, $Pa^{-\nu_i}$

c_i molar concentration of species *i* in gas mixture, mol m^{-3}

c_{i0} molar concentration of species *i* in gas mixture at inlet to adsorber, mol m^{-3}

L length of packed bed adsorber, m

N number of species in the mixture, dimensionless

N_i molar flux of species *i*, mol m^{-2} s^{-1}

p_i partial pressure of species *i* in mixture, Pa

p_t total system pressure, Pa

q_i component molar loading of species *i*, mol kg^{-1}

$\overline{q}_i(t)$ *spatially averaged* component molar loading of species *i*, mol kg^{-1}

r_c radius of crystallite, m

R gas constant, 8.314 J mol^{-1} K^{-1}

t time, s

T absolute temperature, K

u superficial gas velocity in packed bed, m s^{-1}

v interstitial gas velocity in packed bed, m s^{-1}

Greek letters

e voidage of packed bed, dimensionless

r framework density, kg m^{-3}

τ time, dimensionless

Subscripts

i referring to component

i_{break} referring to breakthrough

t referring to total mixture

Section H. Supporting tables

Table S1. Elemental analysis results of $[HO]_{X\%}$-H_2P-COFs and $[HO_2C]_{X\%}$-H_2P-COFs

COFs		C%	H%	N%
$[HO]_{100\%}$-H_2P-COF	Calcd.	76.95	4.25	11.97
	Found	74.92	4.88	10.87
$[HO]_{75\%}$-H_2P-COF	Calcd.	78.29	4.33	12.17
	Found	76.68	4.68	11.54
$[HO]_{50\%}$-H_2P-COF	Calcd.	79.67	4.40	12.39
	Found	78.24	4.87	10.92
$[HO]_{25\%}$-H_2P-COF	Calcd.	81.11	4.48	12.61
	Found	78.62	5.03	11.28
$[HO_2C]_{25\%}$-H_2P-COF	Calcd.	74.05	4.37	10.79
	Found	70.42	4.87	8.67
$[HO_2C]_{50\%}$-H_2P-COF	Calcd.	71.76	4.34	9.85
	Found	69.34	4.71	8.72
$[HO_2C]_{75\%}$-H_2P-COF	Calcd.	69.84	4.31	9.05
	Found	68.46	4.92	7.82
$[HO_2C]_{100\%}$-H_2P-COF	Calcd.	68.21	4.29	8.37
	Found	64.43	5.17	7.02

Table S2. Energy dispersive X-ray spectrometry (EDX) analysis of $[HO]_{X\%}$-H_2P-COFs and $[HO_2C]_{X\%}$-H_2P-COFs

COFs		C%	O%	N%
$[HO]_{100\%}$-H_2P-COF	Calcd.	76.95	6.83	11.97
	Found	77.36	5.92	12.50
$[HO]_{75\%}$-H_2P-COF	Calcd.	78.29	5.21	12.17
	Found	77.2	5.01	13.41
$[HO]_{50\%}$-H_2P-COF	Calcd.	79.67	3.54	12.39
	Found	78.8	3.83	12.91
$[HO]_{25\%}$-H_2P-COF	Calcd.	81.11	1.8	12.61
	Found	82.93	1.81	10.92
$[HO_2C]_{25\%}$-H_2P-COF	Calcd.	74.05	10.79	10.79
	Found	74.50	10.06	11.06
$[HO_2C]_{50\%}$-H_2P-COF	Calcd.	71.76	14.05	9.85
	Found	70.33	14.95	10.38
$[HO_2C]_{75\%}$-H_2P-COF	Calcd.	69.84	16.8	9.05
	Found	68.61	16.98	10.09
$[HO_2C]_{100\%}$-H_2P-COF	Calcd.	68.21	19.13	8.37
	Found	67.08	19.70	8.92

Table S3. Analysis of proton integration of hydrolyzed samples of $[HO_2C]_{X\%}$-H_2P-COFs

COFs	Fragment after hydrolysis	PA 10.25 ppm	DHTA 10.01 ppm	Succinic Acid 2.45 ppm
	structure	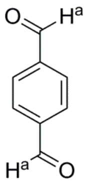	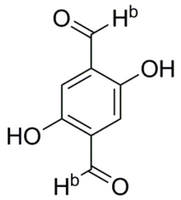	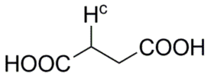
$[HO_2C]_{25\%}$-H_2P-COF	Proton Integration	6.84	2	9.02
	Content of carboxylic acid[a]	= 9.02/4(6.84 + 2) × 100% = 25.5%		
$[HO_2C]_{50\%}$-H_2P-COF	Proton Integration	4.55	4	17.76
	Content of carboxylic acid	= 17.76/4(4.55 + 4) × 100% = 51.9%		
$[HO_2C]_{75\%}$-H_2P-COF	Proton Integration	2.58	6	26.43
	Content of carboxylic acid	= 26.43/4(6 + 2.58) × 100% = 77.1%		
$[HO_2C]_{100\%}$-H_2P-COF	Proton Integration	0	8	33.20
	Content of carboxylic acid	= 33.20/4(8 + 0) × 100% = 103%		

[a]The content of carboxylic acid was calculated by using the equation of $H^c/4(H^a + H^b) \times 100\%$.

The experimentally determined content of carboxylic acid was 25.5%, 51.9%, 77.1%, and 103%, which is consistent with the value of 25%, 50%, 75%, and 100%, calculated from the loading monomer ratios for $[HO_2C]_{25\%}$-H_2P-COF, $[HO_2C]_{50\%}$-H_2P-COF, $[HO_2C]_{75\%}$-H_2P-COF , and $[HO_2C]_{100\%}$-H_2P-COF, respectively.

Table S4. Salient properties of $[HO]_{100\%}$-H_2P-COF and $[HO_2C]_{100\%}$-H_2P-COF (The crystal framework densities, required in the breakthrough simulations, are estimated as (bulk density)/(one-bed porosity) with the assumption that the bed porosity is 0.4.)

COFs	Bulk density (g cm^{-3})	S_{BET} (m^2 g^{-1})	Pore volume (cm^3 g^{-1})	Pore size (nm)
$[HO]_{100\%}$-H_2P-COF	0.24	1186	0.78	2.54
$[HO_2C]_{100\%}$-H_2P-COF	0.26	326	0.49	1.56

Table S5. Langmuir-Freundlich parameters for adsorption of CO_2 in COFs (The experimentally measured excess loadings were first converted to absolute loadings before data fitting.)

COFs	q_{sat} (mol kg^{-1})	b_0 (Pa^{-v})	E (kJ mol^{-1})	n (dimensionless)
$[HO]_{100\%}$-H_2P-COF	2.2	1.4×10^{-11}	34.6	0.9
$[HO_2C]_{100\%}$-H_2P-COF	7.7	6.04×10^{-11}	33.5	0.77

Table S6. One-site Langmuir parameters for N_2 in different materials

COFs	$q_{A,sat}$ (mol kg^{-1})	b_{A0} (Pa^{-1})	E_A (kJ mol^{-1})
$[HO]_{100\%}$-H_2P-COF	1.2	6.14×10^{-12}	30.7
$[HO_2C]_{100\%}$-H_2P-COF	2	2.38×10^{-11}	23.9

Section I. Supporting figures

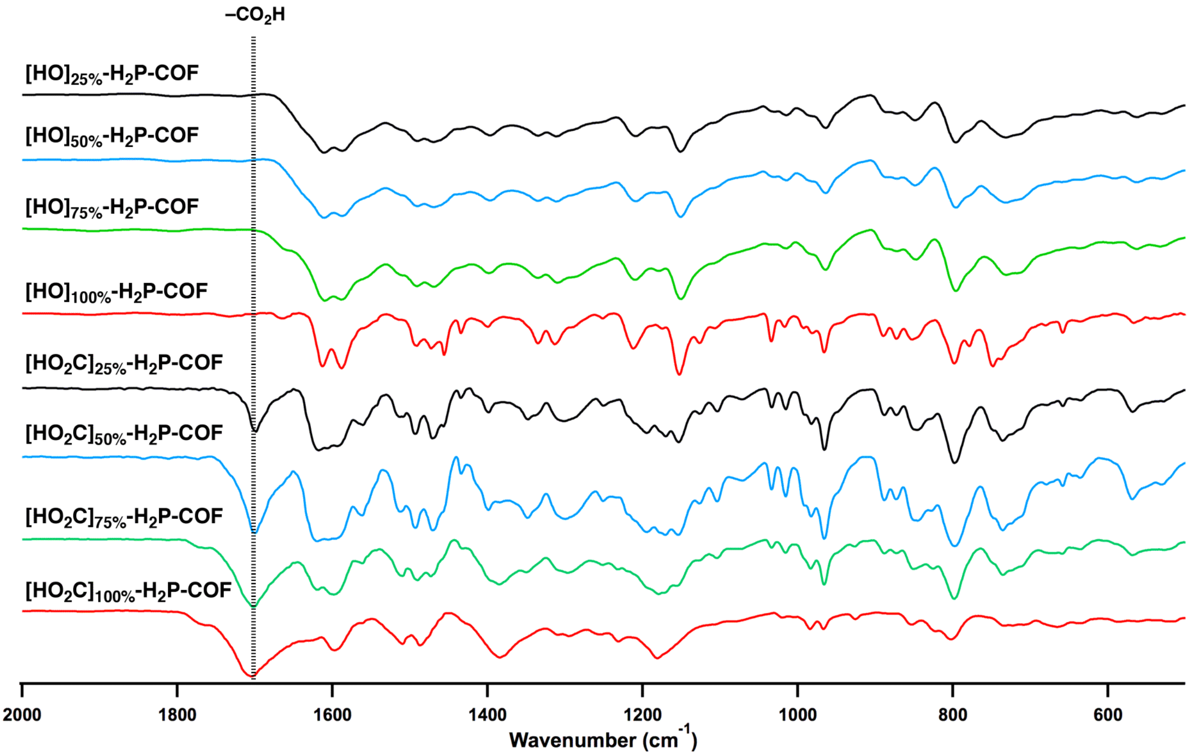

Figure S1. FT IR spectra of $[HO]_{X\%}$-H_2P-COFs and $[HO_2C]_{X\%}$-H_2P-COFs.

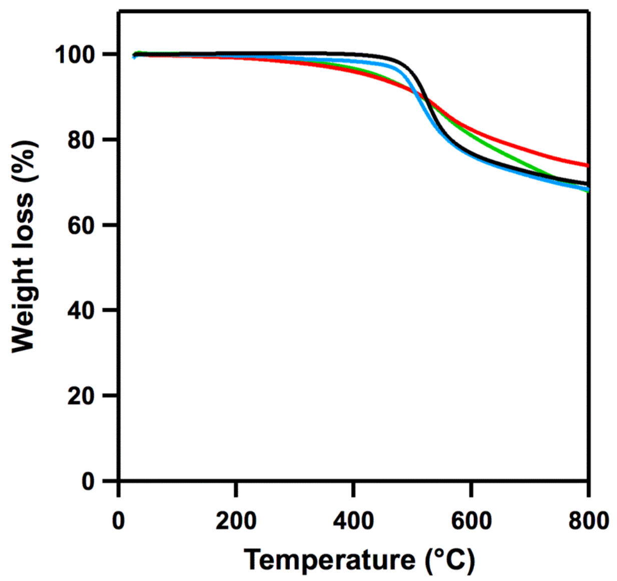

Figure S2. TGA curves of $[HO]_{X\%}$-H_2P-COFs (red: $[HO]_{100\%}$-H_2P-COF, green: $[HO]_{75\%}$-H_2P-COF, blue: $[HO]_{50\%}$-H_2P-COF, black: $[HO]_{25\%}$-H_2P-COF).

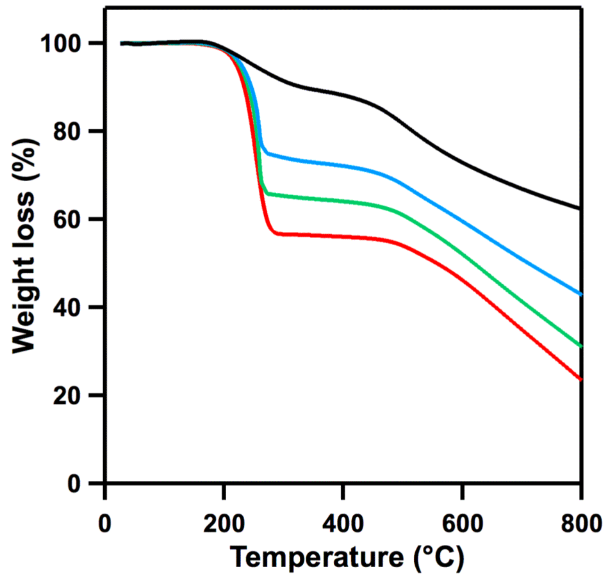

Figure S3. TGA curves of $[HO_2C]_{X\%}$-H_2P-COFs (red: $[HO_2C]_{100\%}$-H_2P-COF, green: $[HO_2C]_{75\%}$-H_2P-COF, blue: $[HO_2C]_{50\%}$-H_2P-COF, black: $[HO_2C]_{25\%}$-H_2P-COF). The weight loss at 250 °C is due to the decomposition of linear chhains on the channel walls and is consistent with the theoretical weight percentage of the chains in the COFs.

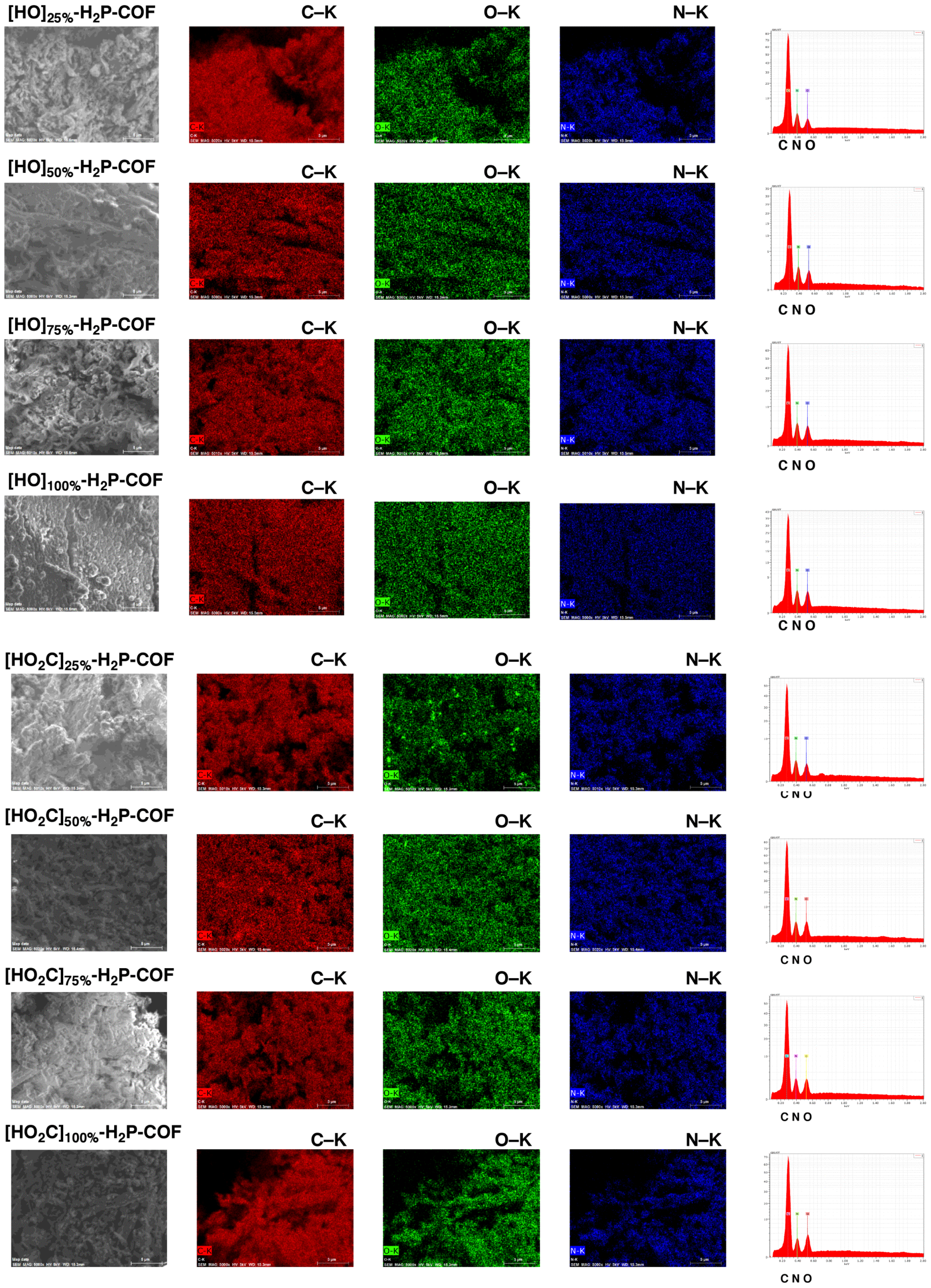

Figure S4. EDX analysis of $[HO]_{X\%}$-H_2P-COFs and $[HO_2C]_{X\%}$-H_2P-COFs.

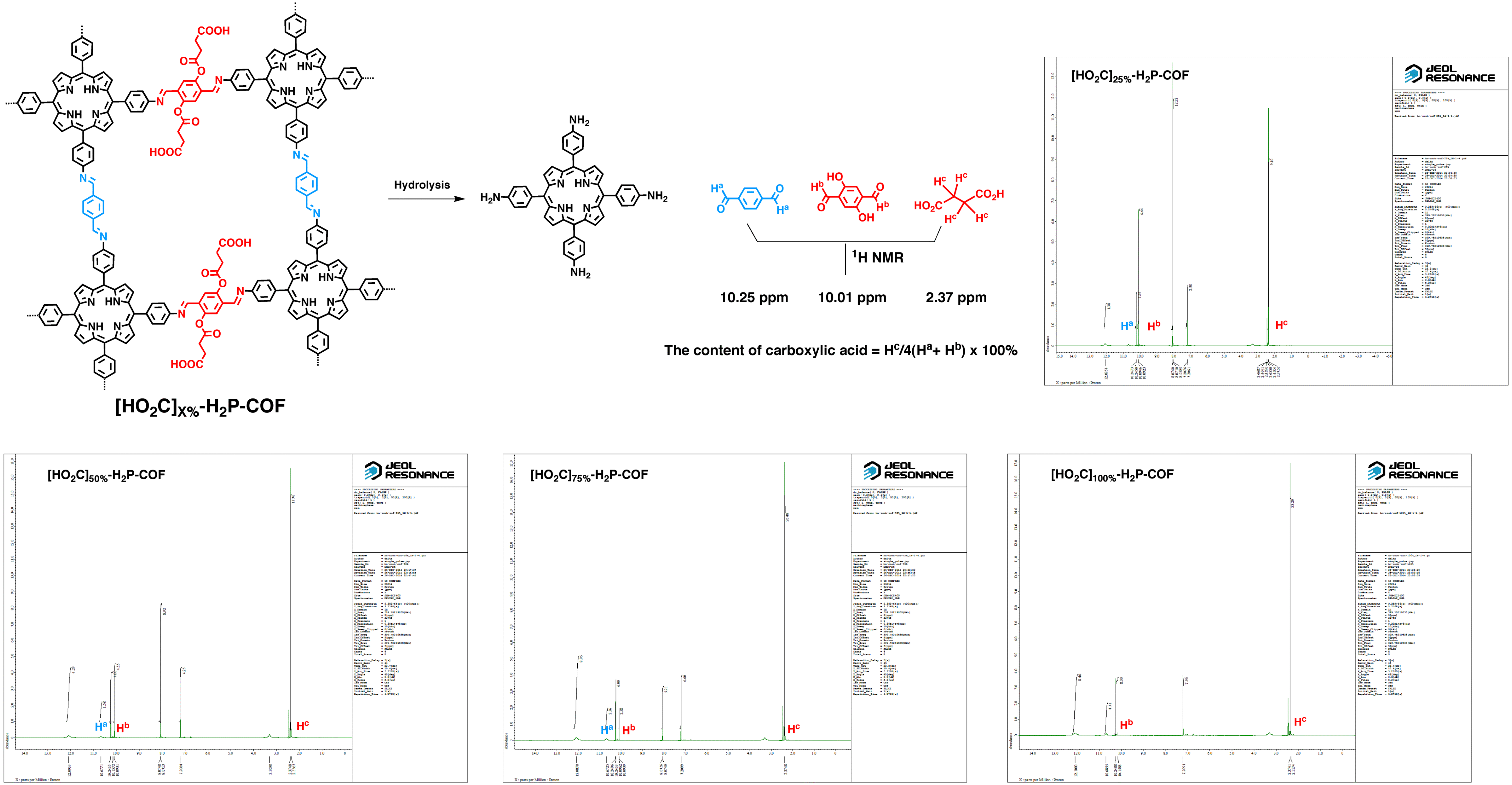

Figure S5. ^{1}H NMR spectra of hydrolyzed samples of $[HO_2C]_{X\%}$-H_2P-COFs.

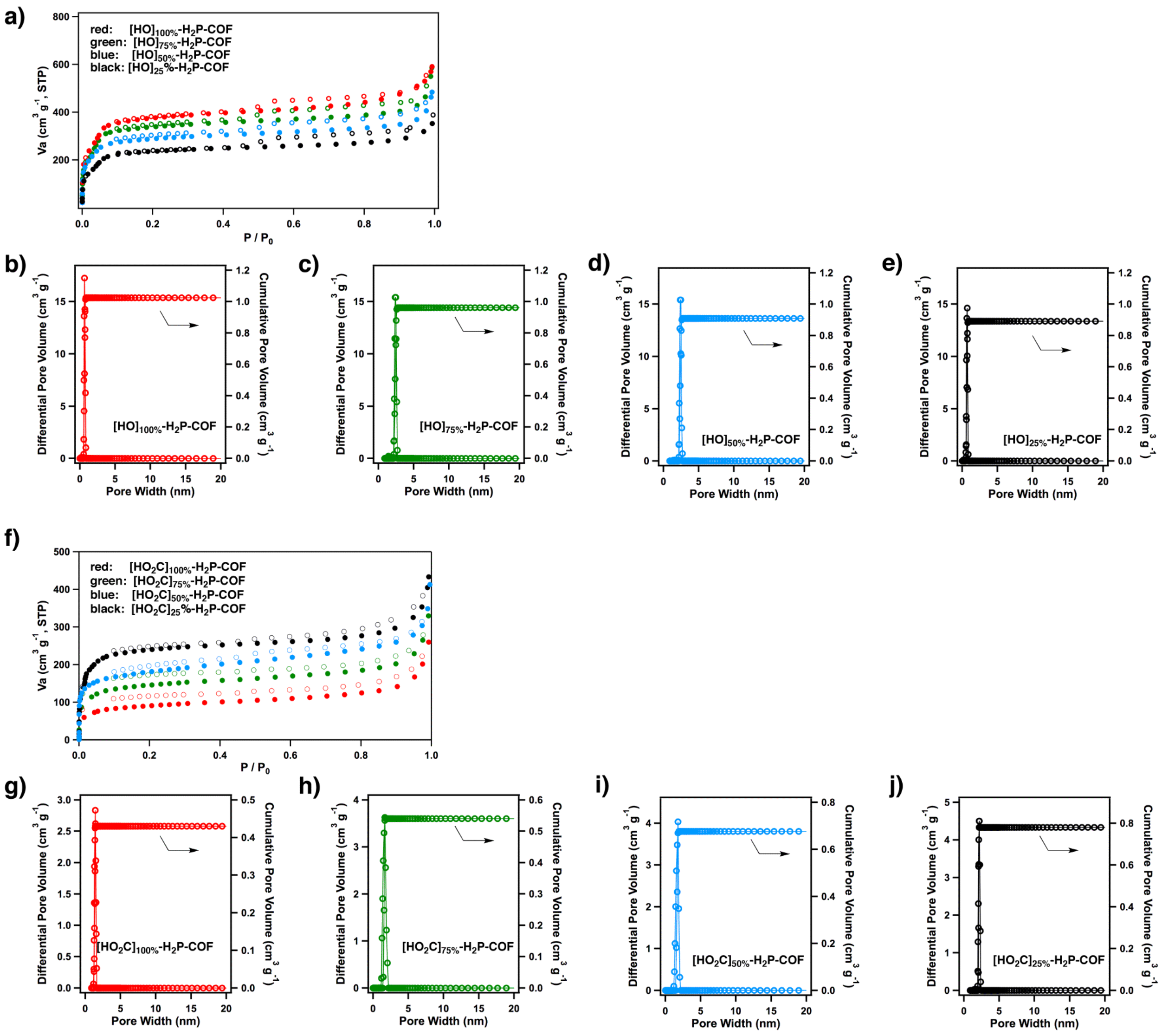

Figure S6. a) Nitrogen sorption isotherm curves of $[HO]_{X\%}$-H_2P-COFs. b-e) Pore size and pore size distribution profiles of $[HO]_{X\%}$-H_2P-COFs. f) Nitrogen sorption isotherm curves of $[HO_2C]_{X\%}$-H_2P-COFs. g-j) Pore size and pore size distribution profiles of $[HO_2C]_{X\%}$-H_2P-COFs.

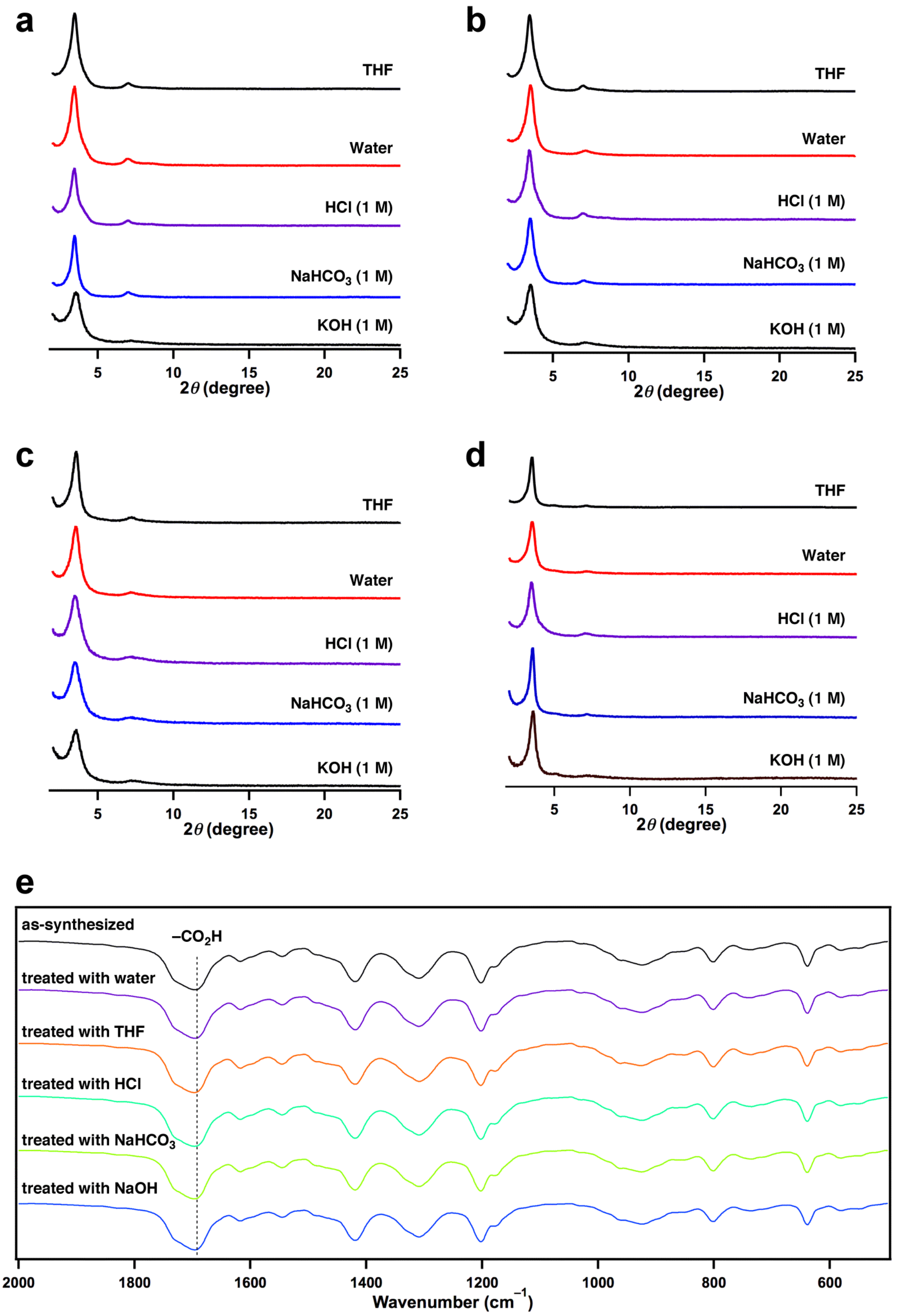

Figure S7. Stability test of $[HO_2C]_{X\%}$-H_2P-COFs. The XRD patterns of a) $[HO_2C]_{25\%}$-H_2P-COF, b) $[HO_2C]_{50\%}$-H_2P-COF, c) $[HO_2C]_{75\%}$-H_2P-COF, and d) $[HO_2C]_{100\%}$-H_2P-COF upon immersion in different solvents at room temperature for 24 h. (e) IR spectra of $[HO_2C]_{100\%}$-H_2P-COF and the $[HO_2C]_{100\%}$-H_2P-COF samples upon treatment with different solvents at room temperature for 24 h. These IR spectra revealed that the carboxylic acid groups were retained under these conditions.

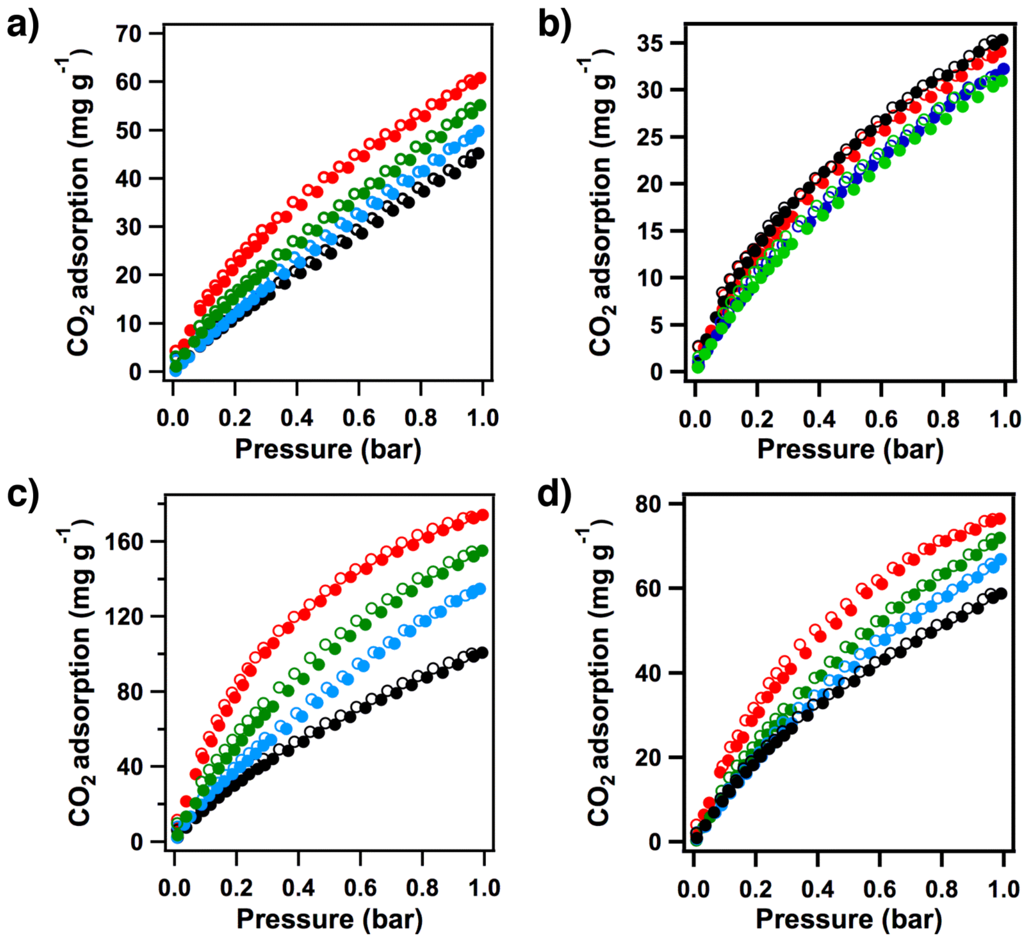

Figure S8. CO_2 sorption curves of $[HO]_{X\%}$-H_2P-COFs measured at (a) 273 K and (b) 298 K (black: $[HO]_{25\%}$-H_2P-COF, blue: $[HO]_{50\%}$-H_2P-COF, green: $[HO]_{75\%}$-H_2P-COF, red: $[HO]_{100\%}$-H_2P-COF). CO_2 sorption curves of $[HO_2C]_{X\%}$-H_2P-COFs measured at (c) 273 K and (d) 298 K (black: $[HO_2C]_{25\%}$-H_2P-COF, blue: $[HO_2C]_{50\%}$-H_2P-COF, green: $[HO_2C]_{75\%}$-H_2P-COF, red: $[HO_2C]_{100\%}$-H_2P-COF). Open and filled circles represent desorption and adsorption, respectively.

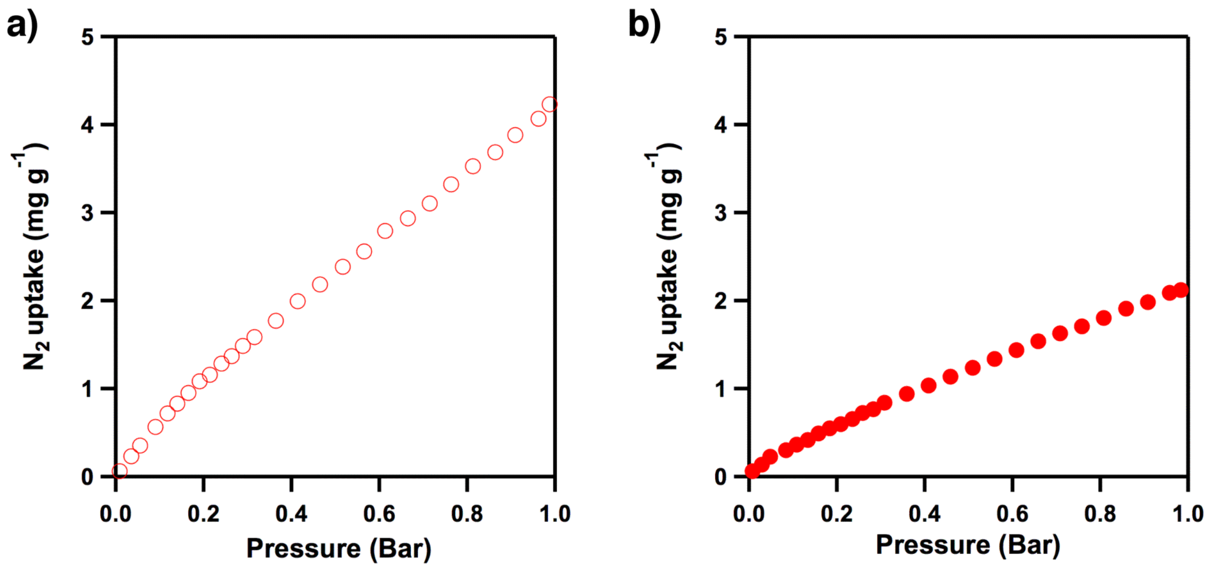

Figure S9. Nitrogen adsorption isotherm curves of a) $[HO]_{100\%}$-H_2P-COF and b) $[HO_2C]_{100\%}$-H_2P-COF measured at 298 K.

Section J. Supporting references

S1. M. Yuasa, K. Oyaizu, A. Yamaguchi, M. Kuwakado *J. Am. Chem. Soc.* **2004**, *126*, 11128.

S2. U. S. Hiremath, *Tetrahedron Letters* **2013**, *54*, 3419.

S3. J. A. Mason, K. Sumida, Z. R. Herm, R. Krishna, J. R. Long, *Energy Environ. Sci.* **2011**, *4*, 3030.

S4. H. Wu, K. Yao, Y. Zhu, B. Li, Z. Shi, R. Krishna, J. Li, *J. Phys. Chem. C* **2012**, *116*, 16609.

S5. A. L. Myers, J. M. Prausnitz, *A.I.Ch.E.J.* **1965**, *11*, 121.

S6. R. Krishna, J. M. van Baten, *Chem. Eng. J.* **2007**, *133*, 121.

S7. R. Krishna, J. M. van Baten, *J. Membr. Sci.* **2010**, *360*, 323.

S8. R. Krishna, J. M. van Baten, *Phys. Chem. Chem. Phys.* **2011**, *13*, 10593.

S9. R. Krishna, J. M. van Baten, *J. Membr. Sci.* **2011**, *383*, 289.

S10. R. Krishna, J. M. van Baten, *J. Membr. Sci.* **2011**, *377*, 249.

S11. R. Krishna, J. M. van Baten, *Chem. Eng. Sci.* **2012**, *69*, 684.

S12. R. Krishna, J. R. Long, *J. Phys. Chem. C* **2011**, *115*, 12941.

S13. R. Krishna, *Microporous Mesoporous Mater.* **2014**, *185*, 30.

S14. R. Krishna, R. Baur, *Sep. Purif. Technol.* **2003**, *33*, 213.
